# LeTetR Positively Regulates 3-Hydroxylation of the Antifungal HSAF and Its Analogs in *Lysobacter enzymogenes* OH11

**DOI:** 10.3390/molecules25102286

**Published:** 2020-05-13

**Authors:** Lingjun Yu, Vimmy Khetrapal, Fengquan Liu, Liangcheng Du

**Affiliations:** 1 Department of Chemistry, University of Nebraska-Lincoln, Lincoln, NE 68588-0304, USA; LingjunYu113@outlook.com (L.Y.); vimmyjagga@gmail.com (V.K.); 2 Institute of Plant Protection, Jiangsu Academy of Agricultural Sciences, Nanjing 210014, China; fqliu20011@sina.com

**Keywords:** natural product, HSAF, alteramides, regulation, *Lysobacter enzymogenes*

## Abstract

The biocontrol agent *Lysobacter enzymogenes* OH11 produces several structurally distinct antibiotic compounds, including the antifungal HSAF (Heat Stable Antifungal Factor) and alteramides, along with their 3-dehydroxyl precursors (3-deOH). We previously showed that the 3-hydroxylation is the final step of the biosynthesis and is also a key structural moiety for the antifungal activity. However, the procedure through which OH11 regulates the 3-hydroxylation is still not clear. In OH11, the gene *orf3232* was predicted to encode a TetR regulator (LeTetR) with unknown function. Here, we deleted *orf3232* and found that the LeTetR mutant produced very little HSAF and alteramides, while the 3-deOH compounds were not significantly affected. The production of HSAF and alteramides was restored in *orf3232*-complemented mutant. qRT-PCR showed that the deletion of *orf3232* impaired the transcription of a putative fatty acid hydroxylase gene, *orf2195*, but did not directly affect the expression of the HSAF biosynthetic gene cluster (*hsaf*). When an enzyme extract from *E. coli* expressing the fatty acid hydroxylase gene, *hsaf*-*orf7*, was added to the LeTetR mutant, the production of HSAF and alteramides increased by 13–14 fold. This study revealed a rare function of the TetR family regulator, which positively controls the final step of the antifungal biosynthesis and thus controls the antifungal activity of the biocontrol agent.

## 1. Introduction

**Figure 1:**
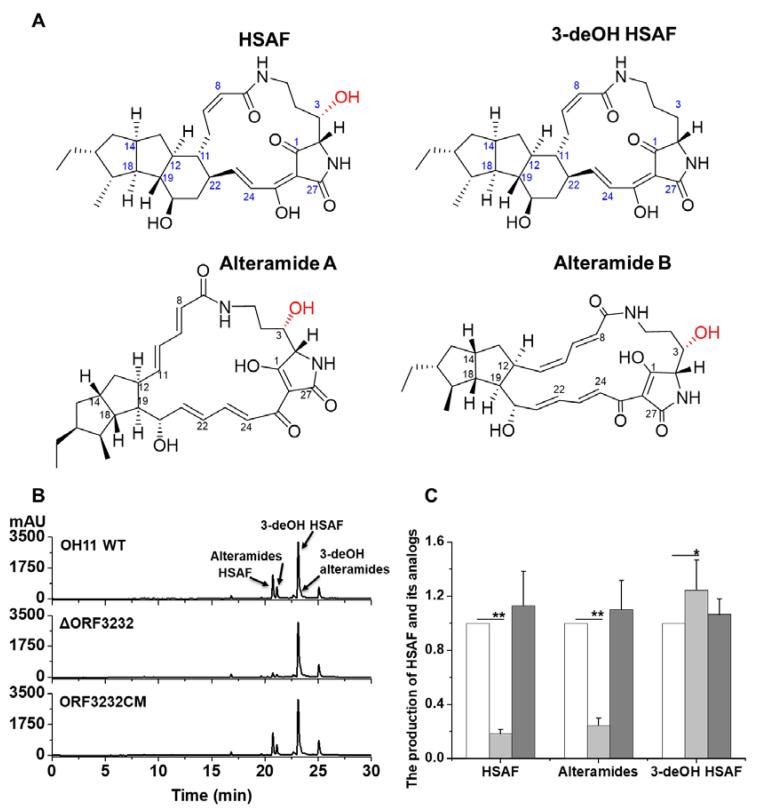
The antifungal HSAF and its analogs and impact of the deletion of the TetR family regulator, LeTetR, on their production in *L. enzymogenes* OH11. (**A**) Chemical structure of HSAF and its analogs produced in *L. enzymogenes* OH11. (**B**) HPLC analysis of HSAF and its analogs in strains WT, ΔORF3232 and ORF3232CM cultured in 1/10 TSB (Tryptic Soy Broth) medium for 48 h. (**C**) Quantification of HSAF and its main analogs in these strains. Note: As the 3-deOH alteramides were minor compounds and not well resolved from 3-deOH HSAF in HPLC, only 3-deOH HSAF was presented in the quantification. White columns, WT; light-gray columns, ΔORF3232; dark-gray columns, ORF3232CM. Data are presented as averages of three independent experiments, each conducted in triplicates, with * *p* < 0.05; ** *p* < 0.01.

*Lysobacter* is a genus of Gram-negative gliding bacteria inhabiting soil and fresh water [1]. Natural products with distinct chemical structures and biological activities have been identified from the *Lysobacter* species that are recognized as a new source of antibiotics (Figure 1A) [2]. HSAF belonging to the family of polycyclic tetramate macrolactams (PoTeM) is a predominate natural product of *Lysobacter enzymogenes* [3,4]. It exhibits a broad-spectrum antifungal activity with a novel mode of action and has the potential as a novel lead compound for fungicides or antifungal drugs [5,6,7].

The regulation of HSAF biosynthesis involves a complex network including two-component regulatory systems (TCS), transcription regulators, and small-molecule signaling factors. Previous studies showed that several signal pathways, such as the TCS Rpf/DSF (diffusible signal factor), DF (diffusible factor), and Clp (cyclic adenosine monophosphate-receptor-like protein), are critical to the biosynthesis of HSAF [8,9,10,11,12,13,14,15,16,17,18]. In the Rpf/DSF pathway, the signal molecule *Le*DSF3 promotes the HSAF production and Clp expression [8]. In the DF pathway, the LysR-family regulator LysR_Le_ binds to the promoter region of HSAF biosynthetic gene and induces the gene expression and HSAF production, after binding with its ligand 4-hydroxybenzoic acid (4-HBA) [11]. In addition, the MarR-family regulator LarR promotes the HSAF biosynthesis by binding to the promoter region of the HSAF biosynthetic gene, and is negatively controlled by 4-HBA but positively controlled by LysR_Le_ [12]. In the Clp pathway, an acetyltransferase Lat that is regulated by Clp is involved in the HSAF biosynthesis [10]. A TetR-family regulator LetR inhibits the HSAF biosynthetic genes expression and HSAF production by binding to the promoter of the biosynthetic gene [13]. The TCS response regulator PilG negatively controls the HSAF production, while another TCS response regulator PilR induces the biosynthesis of HSAF by affecting the in vivo concentration of cyclic di-GMP [14,15]. Cyclic di-GMP regulates the HSAF biosynthesis by controlling the binding of Clp with its binding sites in the HSAF promoter [16] and maintaining a proper cellular concentration of spermidine, which is shown to be critical to the HSAF production [17,18]. Overall, the previous studies mainly focused on the regulation of the final product HSAF of the biosynthetic pathway.

Apart from HSAF, *L. enzymogenes* also produces several PoTeM analogs including alteramides and their 3-dehydroxy forms, 3-deOH HSAF, and 3-deOH alteramides (Figure 1A). Our previous study has shown that 3-hydroxylation is the final step in the biosynthesis of PoTeM, and the fatty acid hydroxylase SD that is encoded by *orf7* of the *hsaf* gene cluster is required for the 3-hydroxylation [19]. The study also indicated that the 3-OH moiety is critical to the antifungal activity of HSAF. However, it remains unclear as to how *L. enzymogenes* controls the production of the active 3-OH compounds versus the barely active 3-deOH compounds. In this work, we found that a new TetR-family regulator, LeTetR encoded by *orf3232* in *L. enzymogenes* OH11, positively regulates the 3-hydroxylation of HSAF and its analogs, but does not affect the production of 3-deOH HSAF and 3-deOH alteramides.

## 2. Results

### 2.1. LeTetR Regulates the Production of HSAF and Alteramides

Genome annotation of *L. enzymogenes* OH11 predicted that *orf3232* encodes a TetR regulator (here named LeTetR). LeTetR contained the DNA-binding domain at the C-terminus and RutR domain, which might be related to the ligand binding at the *N*-terminus (Figure S1). Since the TetR family regulators are known to regulate antibiotics production, we deleted the *orf3232* gene (ΔORF3232) (Figure S2) and examined the impact on the production of the most predominant antibiotics in strain OH11, HSAF, and the analogs (Figure 1A). HPLC analysis showed that the production of HSAF and alteramides in ΔORF3232 decreased significantly, to about 20% of that in the wild type (WT), while the production of 3-deOH HSAF and 3-deOH alteramides increased slightly (Figure 1B,C). When the *orf3232* gene was reintroduced into the mutant, the complementary strain (ORF3232CM) (Figure S3) restored the production of HSAF and alteramides to the WT level, while the level of 3-deOH HSAF and 3-deOH alteramides was nearly unaffected in ORF3232CM (Figure 1B,C).

**Figure 2:**
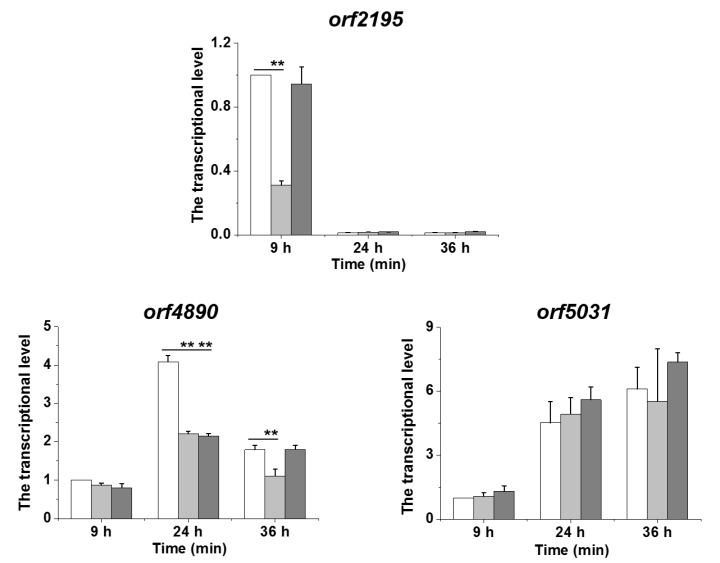
Transcription analysis of fatty acid hydroxylase genes (*orf2195*, *orf4890*, and *orf5031*) in OH11 cultured in 1/10 TSB medium. White columns, WT; light-gray columns, ΔORF3232; and dark-gray columns, ORF3232CM. Data are presented as averages of the three independent experiments, each conducted in triplicates, with ** *p* < 0.01.

The results indicated that LeTetR might participate in the regulation of the 3-hydroxylation of HSAF and alteramides. Our previous study showed that *orf7* (fatty acid hydroxylase gene, or SD gene) in the HSAF biosynthetic gene cluster (*hsaf*) is required for the 3-hydroxylation of HSAF, and *orf8* (ferredoxin reductase gene, or the *FNR* gene) enhanced this process [19]. We thus suspected that LeTetR might regulate the transcription of *orf7* and *orf8* to affect the production of the 3-hydroxyl compounds, HSAF and alteramides. Surprisingly, qRT-PCR results showed that the transcription of *orf7* and *orf8*, as well as the HSAF polyketide-nonribosomal peptide synthetase gene, *hsaf-pks-nrps*, was not significantly changed in the mutant strain ΔORF3232 (Figure S4). This led us to search for other putative fatty acid hydroxylase genes, outside the HSAF biosynthetic gene cluster. Three candidate genes (*orf2195*, *orf4890*, and *orf5031*) were found from the OH11 genome (Figure S5). The transcription of *orf2195* was significantly decreased in the strain ΔORF3232 and restored to the WT level in the strain ORF3232CM, when cultured for 9 h, and then almost could not be detected in all three strains, after 24 h and 36 h (Figure 2). In contrast, the transcription of *orf4890* and *orf5031* did not change significantly between strain ΔORF3232 and ORF3232CM (Figure 2).

**Figure 3:**
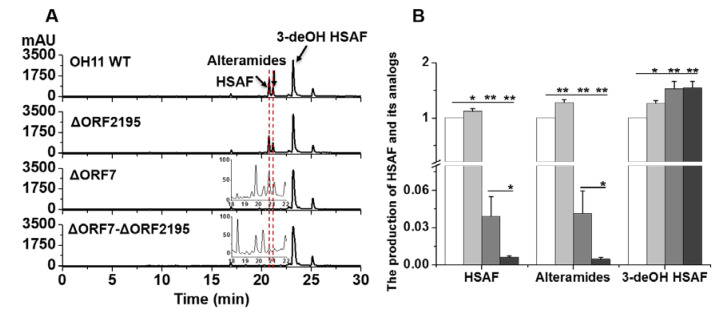
Production of HSAF and its analogs in deletion mutants of fatty acid hydroxylase genes in *L. enzymogenes* OH11. (**A**) HPLC analysis of HSAF and its analogs in strains WT, ΔORF2195, ΔORF7, and ΔORF7-ΔORF2195, cultured in 1/10 TSB medium for 48 h. (**B**) Quantification of HSAF and its analogs in these strains. White columns, WT; light-gray columns, ΔORF2195; medium-gray columns, ΔORF7; dark-grey columns, ΔORF7-ΔORF2195. Note: As the 3-deOH alteramides were minor compounds and not well-resolved from 3-deOH HSAF in HPLC, only 3-deOH HSAF was presented in the quantification. Data are presented as the averages of the three independent experiments, each conducted in triplicates, with * *p* < 0.05; ** *p* < 0.01.

The result suggested that the LeTetR positively regulated the transcription of *orf2195*, and ORF2195 in turn might also contribute to the conversion of 3-deOH HSAF and 3-deOH alteramides to HSAF and alteramides. To test this hypothesis, we deleted *orf2195* (ΔORF2195), as well as *orf7* (ΔORF7) (Figures S6 and S7) and analyzed the compounds in the deletion mutants. The HPLC results showed that the level of the compounds in strain ΔORF2195 was similar to that in WT, indicating *orf2195* alone did not play a significant role in the 3-hydroxylation (Figure 3). However, the level of HSAF and alteramides in strain ΔORF7 was significantly reduced, while the level of 3-deOH HSAF (also probably 3-deOH alteramides) increased slightly (Figure 3). The result is consistent with the previous study [19] and revealed that, while ORF7 (SD) is the main enzyme for 3-hydroxylation, other fatty acid hydroxylases, such as ORF2195, are also likely to contribute to the conversion of 3-deOH compounds to HSAF and alteramides (Figure 3). Finally, we generated a double deletion mutant (ΔORF7-ORF2195), by deleting both *orf2195* and *orf7* (Figure S8). The level of HSAF and alteramides in strain ΔORF7-ORF2195 further decreased when compared to that of strain ΔORF7 or strain ORF2195 (Figure 3), supporting that ORF2195 plays a role in HSAF 3-hydroxylation.

### 2.2. Bioconversion of 3-deOH Compounds in the LeTetR Mutant to HSAF and Alteramides Using an Enzyme Extract from the E. coli-Expressing orf7 Gene

**Figure 4:**
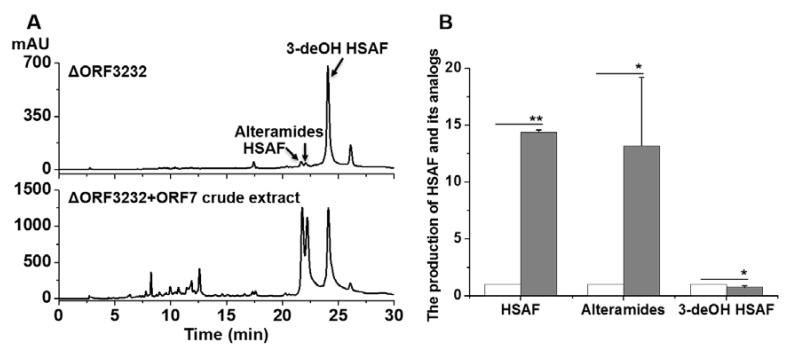
Bioconversion of 3-deOH compounds to HSAF and alteramides. (**A**) HPLC analysis of HSAF and its analogs in the LeTetR mutant, ΔORF3232, with or without addition of an enzyme extract from the *E. coli*-expressing *hsaf-orf7*. ΔORF3232 was cultured in 1/10 TSB medium for 72 h. (**B**) Quantification of HSAF and its analogs in the cultures. Note: As the 3-deOH alteramides were minor compounds and not well-resolved from 3-deOH HSAF in HPLC, only 3-deOH HSAF was presented in the quantification. White columns, ΔORF3232 without the enzyme extract; gray columns, ΔORF3232 with the enzyme extract. Data are presented as averages of three independent experiments, each conducted in triplicates, with * *p* < 0.05; ** *p* < 0.01.

The above results confirmed that the fatty acid hydroxylase encoded by *orf7* (SD gene) in the HSAF biosynthetic gene cluster is the main player in the 3-hydroxylation of HSAF. This inspired us to test the bioconversion of the 3-deOH compounds produced in the LeTetR mutant, ΔORF3232, using the *E. coli*-carrying *hsaf-orf7* gene. When the *E. coli* extract was added to the *Lysobacter* culture of ΔORF3232, HSAF and alteramides dramatically increased (14-fold and 13-fold, respectively) in the ΔORF3232 culture. In addition, the 3-deOH compounds remained at a high level in ΔORF3232, with the addition of the *E. coli* enzyme extract (Figure 4). This showed that the *Lysobacter* strain ΔORF3232 continually produced the 3-deOH compounds, while a large portion of the 3-deOH compounds were converted to HSAF and alteramides by the *E. coli*-expressed enzyme. The results confirmed the 3-hydroxylation function of *hsaf-orf7* and also revealed a potentially robust bioconversion for production of the active PoTeM compounds (HSAF and alteramides), by using the LeTetR mutant as a cell factory.

## 3. Discussion

The TetR family regulators are typically repressor proteins that negatively control the gene expression in many cellular processes, such as antibiotic resistance, biosynthesis, pathogenesis, and response to cell stress [20]. In this study, we investigated the function of a TetR regulator (LeTetR) encoded by *orf3232* in *L. enzymogenes* OH11. When this gene was deleted, the production of the 3-hydroxyl products, HSAF and alteramides, was significantly reduced, while the production of their biosynthetic precursors, 3-deOH HSAF and 3-deOH alteramides, was not apparently affected (Figure 1). This shows that LeTetR positively regulates the 3-hydroxylation step in the production of this family of antifungal natural products. It also suggests that LeTetR might selectively control the final step in the biosynthesis, which is key to the conversion of the barely active precursors, the 3-deOH compounds, to the highly active final products—HSAF and alteramides. Wang et al. previously studied a TetR regulator (LetR) in OH11, which is encoded by *orf0953* [13]. Their results showed that LetR negatively regulated HSAF production, through a transcription repression by directly binding to the promoter (P_HSAF_) of the HSAF biosynthetic gene cluster. The LetR-regulated HSAF biosynthesis was due to the direct control of the transcription of the biosynthetic gene (including the key biosynthetic gene *hsaf-pks-nrps*). However, our results show that LeTetR did not appear to directly affect the transcription of *hsaf-pks-nrps*, or the associated genes in the cluster, such as *orf7* (SD gene) and *orf8* (FNR gene) for 3-hydroxylation (Figure S4). In search for other putative fatty acid hydroxylase genes that might be controlled by LeTetR and so might contribute to the 3-hydroxylation, we found the transcription of one gene, *orf2195*, was clearly impaired upon deletion and restored upon complementation of the regulator LeTetR gene, *orf3232* (Figure 2). However, *orf2195* appeared to play a minor role in the 3-hydroxylation, because the deletion of *orf2195* only had a minor effect on the 3-hydroxyl products. When both *orf2195* and *orf7* were deleted, the 3-hydroxyl products were barely detectable, while the accumulation of the 3-deOH products increased in the double deletion mutant (Figure 3). Together, the results revealed an unusual TetR type regulator that positively controlled the final step of the HSAF biosynthetic pathway.

Despite the interesting findings, it should be pointed out that the exact mechanism by which LeTetR selectively controls the 3-hydroxylation step in the biosynthesis of HSAF is not completely understood. The results showed that the transcription level of the *orf7* gene was not affected in the *orf3232* mutant ΔORF3232, which mainly produced the 3-deOH precursors, with very little final products (HSAF and alteramides). However, ORF7 is the main enzyme to convert the 3-deOH precursors to the final products. The underlying mechanism for this observation is unclear, but one possibility could be that *orf3232* might regulate the translation or catalytic activity of the ORF7 enzyme. The decreased level of HSAF and alteramides in the *orf3232* mutant might be due to a decrease of the functional enzymes, which was rescued by the *E. coli*-produced ORF7. More studies are needed to tackle this fairly unusual LeTetR encoded by *orf3232*, which could reveal new insights into the function of the TetR family regulators. Nevertheless, our results showed that the strain ΔORF3232 could be used as a cell factory to generate the 3-deOH compounds, which are inactive precursors of the final antifungal compounds, HSAF and alteramides. The wild-type OH11 produced a complex of HSAF and analogs, which are controlled by a network of regulators and signals [8,9,10,11,12,13,14,15,16,17,18]. Small changes in culture conditions could affect the production of the compounds. In contrast, strain ΔORF3232 produced a relatively simple profile of compounds. Moreover, after the ORF7 extract was added to the strain ΔORF3232, the yield of the 3-deOH precursors decreased only slightly (~75% of that without ORF7 extract), while the yield of HSAF and alteramides increased dramatically (14-fold and 13-fold, respectively) (Figure 4). This indicated that exogenous ORF7 could not only convert the 3-deOH precursors to the final compounds, but could also drive the strain ΔORF3232 to continuously produce these precursors. Thus, the overall yield of HSAF and alteramides could be significantly enhanced using this method.

## 4. Materials and Methods

### 4.1. Bacterial Strains, Plasmids, and Growth Conditions

The bacterial strains and plasmids used in this study are shown in Table S1. The Luria–Bertani (LB) broth medium was used for the growth of *L. enzymogenes* OH11 (CGMCC No. 1978). 1/10 TSB (3 g TSB, per liter) was used for the RNA extraction and HSAF production. Plasmid pJQ200SK was used for gene deletion and complementary in *L. enzymogenes* OH11 [21]. The *Escherichia coli* strain XL-1 Blue was cultured at 37 °C in the LB medium supplemented with gentamicin (Gm, 50 g/mL) to propagate plasmids. *E. coli* strain S17 was used for intergeneric conjugation. *E. coli* strain BL21 (DE3)/ORF7 was used for the ORF7 expression.

### 4.2. DNA Manipulation and Lysobacter Transformation

Chromosomal DNA and plasmids were isolated from *L. enzymogenes* OH11 or *E. coli*, according to the standard techniques. Database search and sequence analysis were performed using the online program PSI-BLAST. For the *Lysobacter* transformation, the recombination plasmid was introduced into *L. enzymogenes* OH11 through intergeneric conjugation, using *E. coli* strain S17. After growing on LB plates with Kanamycin (Km, 100 μg/mL) and Gentamicin (Gm, 150 μg/mL) at 30 °C for 72 h, the transformants were plated on the LB plates supplemented with 10% (*w*/*v*) sucrose and Km (100 μg/mL). After growing at 30 °C for 72 h, the colonies were transferred into the LB plates containing Km (100 μg/mL) or Km (100 μg/mL) + Gm (150 μg/mL). Finally, the Km-resistant and Gm-sensitive strains were selected for PCR verification.

### 4.3. Primers and PCR

All primers used in this study were listed in Table S2. Phusion High-Fidelity DNA polymerase (Thermo Scientific, Waltham, MA, USA) and Taq DNA polymerase (Thermo Scientific, Waltham, MA, USA) were used for PCR program. For Phusion DNA polymerase, an initial denaturation at 98 °C for 30 s was followed by 30 cycles of amplification (98 °C for 10 s, 60 °C for 15 s, 72 °C for 1 min), and additional 5 min at 72 °C. For Taq DNA polymerase, an initial denaturation at 95 °C for 3 min was followed by 30 cycles of amplification (95 °C for 30 s, 60 °C for 30 s, 72 °C for 1 min), and an additional 10 min at 72 °C. Considering different DNA templates and primers, the annealing temperature and the elongation time were changed in some cases.

### 4.4. Generation of the Deletion Mutant of orf3232 and Its Complementary Strain

The DNA fragments corresponding to the upstream and downstream region of *orf3232* were amplified using ORF3232UF/UR and ORF3232DF/DR. After treating the upstream region with *Xho*I/*Spe*I, and treating the downstream region with *Bam*HI/*Xho*I, the two DNA fragments were ligated into the *Bam*HI/*Spe*I sites of the plasmid pJQ200SK, to generate the recombination plasmid pJQ200SK::ORF3232. The plasmid was introduced into *L. enzymogenes* OH11 through intergeneric conjugation. After antibiotic and sucrose screening, the transformants were used for PCR verification by primers ORF3232DF/UR and ORF3232VFI/VRI (Figure S2).

For complementary analysis, the DNA fragment containing the upstream region, the downstream region, and the *orf3232* gene was amplified using primers ORF3232CF/UR. Then, the fragment was treated with *Xho*I/*Spe*I and ligated into the same sites of plasmid pJQ200SK, to generate the recombination plasmid pJQ200SK::ORF3232C. The recombination plasmid was transferred into the ΔORF3232 strain through intergeneric conjugation. After antibiotic and sucrose screening, the transformants were used for PCR verification using primers ORF3232DF/UR and ORF3232VFI/VRI (Figure S3).

### 4.5. Construction of the Deletion Mutant of Fatty Acid Hydroxylase Genes

The DNA fragments corresponding to the upstream and downstream region of the fatty acid hydroxylase genes (*orf2195* or *orf7* in HSAF biosynthetic gene cluster) were amplified using the primers listed in Table S2. After treating with the restriction enzymes, the two DNA fragments were ligated into the *Apa*I/*Spe*I sites of plasmid pJQ200SK, to generate the recombination plasmid pJQ200SK::ORF2195 or pJQ200SK::ORF7. The plasmid was introduced into *L. enzymogenes* OH11 through intergeneric conjugation. After antibiotic and sucrose screening, the transformants were used for PCR verification through the primers listed in Table S2 (Figures S6 and S7). To construct the double deletion mutant *orf7* and *orf2195*, the plasmid pJQ200SK::ORF2195 was introduced into ΔORF7 strain through intergeneric conjugation. After antibiotic and sucrose screening, the transformants were used for PCR verification by the primers listed in Table S2 (Figure S8).

### 4.6. RNA Extraction and Real-Time PCR

RNA was isolated from the OH11 WT, ΔORF3232, and ORF3232CM strains cultured in 1/10 TSB medium for 9, 24, and 36 h, as described previously [22]. After removing the contaminated DNA using DNase I (Thermo Scientific, Waltham, MA, USA), RNA was reverse transcribed to the complementary DNA using SuperScript^TM^ II RT reagent kit (Invitrogen, Carlsbad, CA, USA). Real-time PCR was carried out in the CFX Connect Real-Time PCR Detection System (BIO-RAD Laboratories, Inc, Hercules, CA, USA) using PowerUp SYBR Green Master Mix (Applied Biosystems, Foster, CA, USA), using the primers listed in Table S2. The conditions were used as follows—50 °C for 2 min, 95 °C for 2 min, followed by 40 cycles of 95 °C for 15 s and 60 °C for 1 min. The 16S rRNA was used as an internal control. The relative transcriptional levels of the tested genes were normalized to 16S rRNA and determined using the 2^−△△CT^ method [23].

### 4.7. Extraction and HPLC Analysis of HSAF and Its Analogs

OH11 WT, ΔORF3232, and ORF3232CM strains were incubated into 1 mL LB at 30 °C with overnight shaking at 200 rpm. An aliquot (1%) of the cultures was transferred to 25 mL 1/10 TSB, at 30 °C with shaking at 200 rpm, for 48 h. The culture broths were treated with 75 μL formic acid and 25 mL ethyl acetate. The organic phase was dried with the air flow, and the residues were re-dissolved in 200 μL methanol. A 20 μL aliquot of each extract was analyzed by HPLC (Agilent, 1220 Infinity LC, Santa Clara, CA, USA). Water/0.05% formic acid (solvent A) and acetonitrile/0.05% formic acid (solvent B) were used as the mobile phases with a flow rate of 1.0 mL/min. The HPLC program was as follows—5−25% B in 0−5 min, 25–80% B in 5−25 min, 80−100% B in 25−26 min, maintained to 28 min, back to 5% B at 29 min, and maintained to 30 min. HSAF and its analogs were detected at 318 nm on a UV detector.

### 4.8. Preparation of Protein Crude Extract Containing ORF7

The strain BL21 (DE3)/ORF7 [19] was incubated into LB containing 50 µg/mL kanamycin, and the culture was grown at 37 °C with shaking at 250 rpm. When the optical density (OD_600_) of the culture reached 0.6–0.7, the protein expression was induced with 1 mM IPTG. The cells were allowed to further grow for 15 h at 16 °C. Finally, the cells were harvested through centrifugation at 4 °C and resuspended in PBS (0.24 g KH_2_PO_4_, 1.44 g Na_2_HPO_4_, 8 g NaCl, 0.2 g KCl, pH 7.5, per liter), followed by sonication and centrifugation. The supernatant containing ORF7 was used as the enzyme crude extract.

### 4.9. Exogenous Addition of the ORF7 Crude Extract to the ΔORF3232 Strain

ΔORF3232 strain was incubated into 4 mL LB at 30 °C with overnight shaking at 200 rpm. An aliquot (1%) of the cultures was transferred to 25 mL 1/10 TSB at 30 °C, with shaking at 200 rpm for 48 h. After this, the ORF7 crude extract was added into the cultures, and incubated at 30 °C with shaking at 200 rpm for 24 h. Then, the production of HSAF and its analogs was extracted and analyzed using the above-mentioned method.

## Supplementary Materials

The following are available online. Table S1: Bacterial strains and plasmids used in this study. Table S2: Primers used in this study. Figure S1: The conserved domains in ORF3232. Figure S2: Deletion of *orf3232*. Figure S3: Complementary strain of ΔORF3232. Figure S4: Transcriptional analysis of the HSAF biosynthetic genes, *pks-nrps*, *orf7* and *orf8*. Figure S5: Alignment of the amino acid sequences of the putative fatty acid hydroxylases in OH11. Figure S6: Deletion of *orf2195*. Figure S7: Deletion of *orf7* of the HSAF biosynthetic gene cluster. Figure S8: Deletion of both *orf7* and *orf2195*.
