# Supplementary material for: LeTetR Positively Regulates 3-Hydroxylation of the Antifungal HSAF and Its Analogs in Lysobacter enzymogenes OH11
Source: Molecules. 2020 May 13;25(10):2286. doi: 10.3390/molecules25102286 (PMC7287984; doi:10.3390/molecules25102286)
Supplement: Supplementary file 1 [file molecules-25-02286-s001.pdf]

Supporting Information

LeTetR positively regulates 3-hydroxylation of the antifungal HSAF and its analogs in *Lysobacter enzymogenes* OH11

Lingjun Yu [1,2,#], Vimmy Khetrapal[1,#], Fengquan Liu[2] and Liangcheng Du[1,*]

[1]Department of Chemistry, University of Nebraska-Lincoln, Lincoln, NE, 68588-0304, USA.

[2]Institute of Plant Protection, Jiangsu Academy of Agricultural Sciences, Nanjing, 210014, China

*Correspondences: ldu3@unl.edu, Tel.: + 1-402-472-2998.

[#] These authors contributed equally

Table S1. Bacterial strains and plasmids used in this study

Bacterial strains/plasmids	Relevant characteristics[a]	Source/references
***Lysobacter enzymogenes* OH11**		
OH11	Wild-type, Km^r	(Qian et al., 2009)
ΔORF3232	The deletion mutant of *orf3232* in OH11	This study
ORF3232CM	The complementary strain of ΔORF3232	This study
ΔORF7	The deletion mutant of *orf7* of HSAF biosynthetic gene cluster in OH11	This study
ΔORF2195	The deletion mutant of *orf2195* in OH11	This study
ΔORF7-ΔORF2195	The double deletion mutant of *orf7* and *orf2195* in OH11	This study
**Other bacteria**		
Escherichia coli strain		
XL-1 Blue	Host strain for molecular cloning	Laboratory collection
S17-1	Strain for conjugation with *Lysobacter*	Laboratory collection
BL21 (DE3)/ORF7	Strain for ORF7 expression	(Li *et al.*, 2012)
**Plasmids**		
pJQ200SK	Cloning vector, Gm^r	(Quandt and Hynes 1993)
pJQ200SK::ORF3232	Plasmid for the deletion of *orf3232*, Gm^r	This study
pJQ200SK::ORF3232C	Plasmid for the complementary of *orf3232*, Gm^r	This study
pJQ200SK::ORF7	Plasmid for the deletion of *orf7* of HSAF biosynthetic gene cluster, Gm^r	This study
pJQ200SK::ORF2195	Plasmid for the deletion of *orf2195*, Gm^r	This study

[a]Km^r, Kanamycin resistant; Gm^r, Gentamicin resistant

Table S2. Primers used in this study

Primer	Sequence (5'-3')
ORF3232UF	CCGCTCGAGCATGACGGCAAGGTTACA (*Xho*I)
ORF3232UR	GGACTAGTGTTTCCAGCAAGGTCACG (*Spe*I)
ORF3232DF	CGGGATCCCGCCTGGGTGGGATTGAG (*Bam*HI)
ORF3232DR	CCGCTCGAGTTGTTGACCGAGGCCCAG (*Xho*I)
ORF3232CF	CCGCTCGAGCGCCTGGGTGGGATTGAG (*Xho*I)
ORF3232VFI	CGGGTCGTCGGTGTTGAG
ORF3232VRI	CGCACCGTCTACAACCACTT
ORF7UF	CGGGATCCCAGCAACACGAAGGCGAA(*Bam*HI)
ORF7UR	GGACTAGTAGGCTGTCGTTCCAATGC(*Spe*I)
ORF7DF	CGGGGCCCGGAAATCGAAGCAGTAGG(*Apa*I)
ORF7DR	CGGGATCCCTGCTGTCCACCT TGTTG(*Bam*HI)
ORF7VFI	TTCGCCTTCGTGTTGCTG
ORF7VRI	GGGCGATGACGAAGATGC
ORF2195UF	CGGGGCCCCCAGGGCATCAAGGTCCA(*Apa*I)
ORF2195UR	CGGGATCCCCCGAGCAGGAA CGAGAA(*Bam*HI)
ORF2195DF	CGGGATCCCAGACCCGCTCGGTGCTC(*Bam*HI)
ORF2195DR	GGACTAGTCACGGTCACAGCACGCAC(*Spe*I)
ORF2195VFI	TCCCTACGACACCGACTGG
ORF2195VRI	ATTGGCGTGGGCGATGTA
pks-nrps-real-F	CGGACTCCAGCAAGAAGC
pks-nrps-real-R	GATGATGGCGATGCGGTC
orf7-real-F	CTTCGCCTTCGTGTTGCT
orf7-real-R	GACCACGGTGAGGAAGAAG
orf8-real-F	GCATTGGAACGACAGCCTC
orf8-real-R	GATCGAGTAGGCGCGCAT
ORF3232-real-F	CAACCACTTCCCGAGCAA
ORF3232-real-R	GCCAGGTCGATGAAGGATT

ORF2195-real-F	CCCTACGACACCGACTGG
ORF2195-real-R	CATCAATGCCAGCGGAAT
ORF4890-real-F	CGGAACGGCTGAACCTGT
ORF4890-real-R	GGACGAAGAACTGGAAGGC
ORF5031-real-F	CTCGGTCAACTACGCCCAC
ORF5031-real-R	CGCCGAGATGGATGAACT
OH11-16S-real-F	GTGCGTAGGTGGTTTGTTAA
OH11-16S-real-R	ATCTAATCCTGTTTGCTCCC

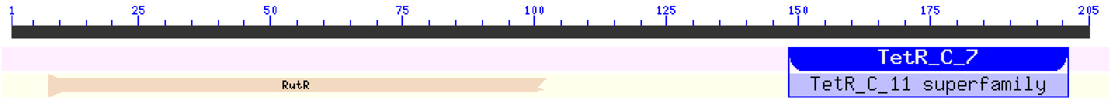

Figure S1. The conserved domains in ORF3232. RutR, pyrimidine utilization regulatory protein R domain.

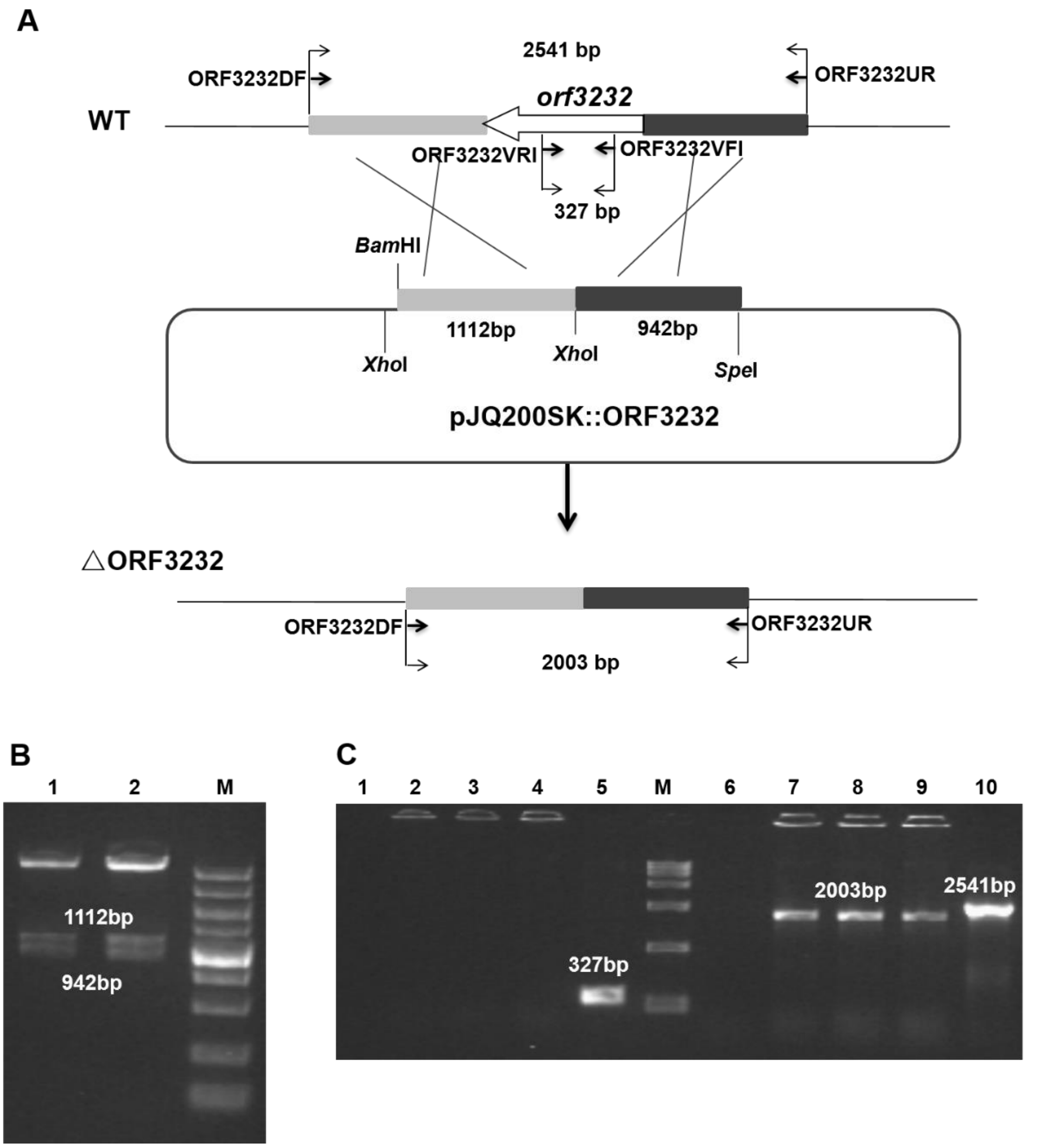

Figure S2. Deletion of *orf3232*. (A) Schematic representation of the *orf3232* deletion in WT strain. (B) Verification of the plasmid pJQ200SK::ORF3232. 1, 2: pJQ200SK::ORF3232 treated with *Xho*I/*Spe*I, and the products of 1112 bp and 942 bp were expected; M: DNA marker. (C) PCR verification of ΔORF3232 strain. 1, 6: negative control, with H_2O used as the template; 2-4: the deletion mutant strains using primers ORF3232VFI/VRI; 5: positive control, with the gDNA of WT used as the template by primers ORF3232VFI/VRI, and the product of 327 bp was expected; 7-9: the deletion mutant strains using primers ORF3232DF/UR, and the product of 2003 bp was expected; 10: positive control, with the gDNA of WT used as the template by primers ORF3232DF/UR, and the product of 2541 bp was expected; M: DNA marker.

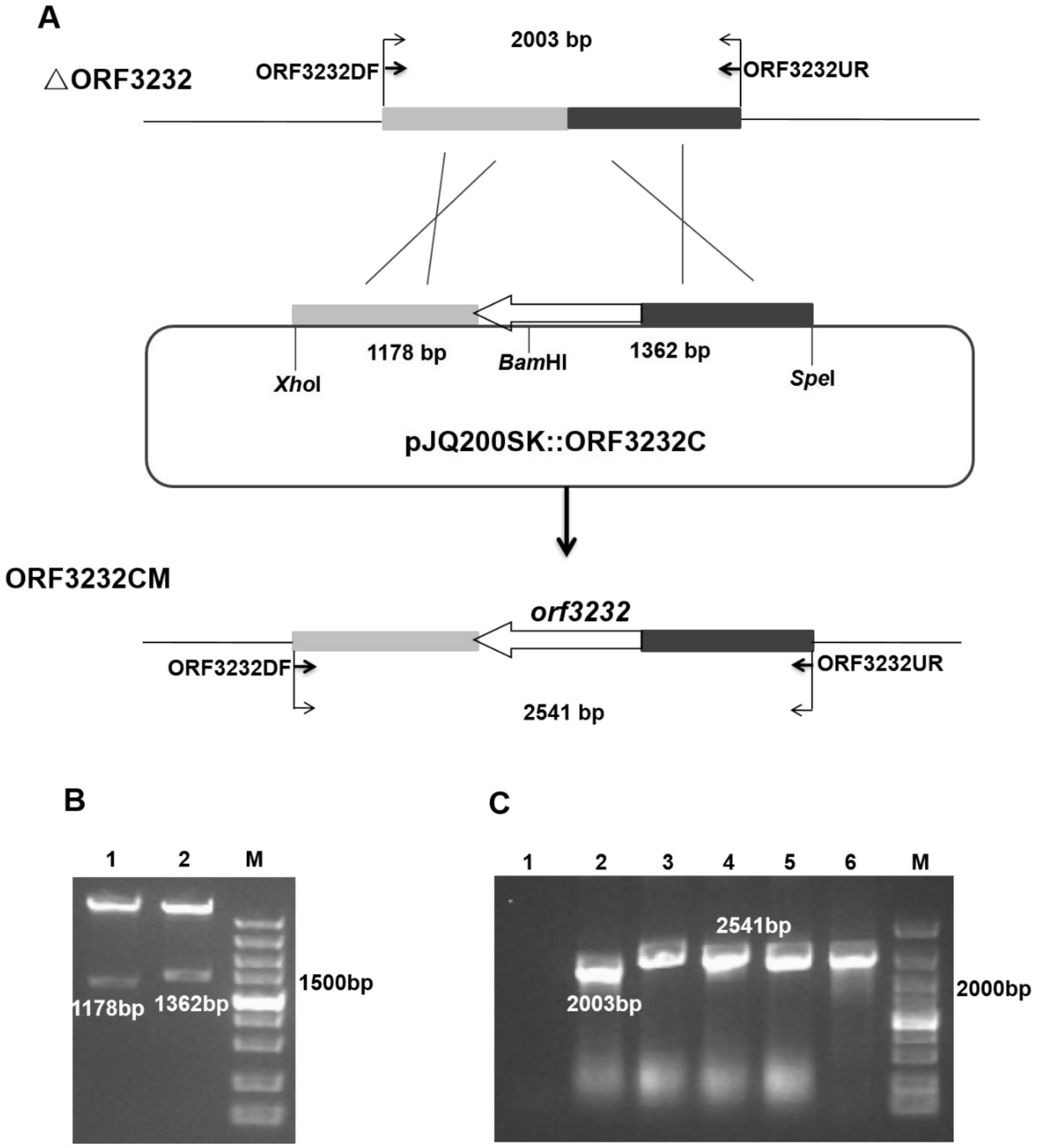

Figure S3. Complementary strain of ΔORF3232. (A) Schematic representation of *orf3232* complementary in ΔORF3232 strain. (B) Verification of the plasmid pJQ200SK::ORF3232C. 1: pJQ200SK::ORF3232C treated with *Xho*I/*Bam*HI, and the product of 1178 bp was expected; 2: pJQ200SK::ORF3232C treated with *Bam*HI/*Spe*I; the product of 1362 bp was expected. (C) PCR verification of ORF3232C strain. 1: negative control, with H_2O used as the template; 2: the deletion mutant strains using primers ORF3232DF/UR, and the product of 2003 bp was expected; 3-5: the ORF3232C strains using primers ORF3232DF/UR, and the product of 2541 bp was expected 6: positive control, with the gDNA of WT used as the template by primers ORF3232DF/UR, and the product of 2541 bp was expected; M: DNA marker.

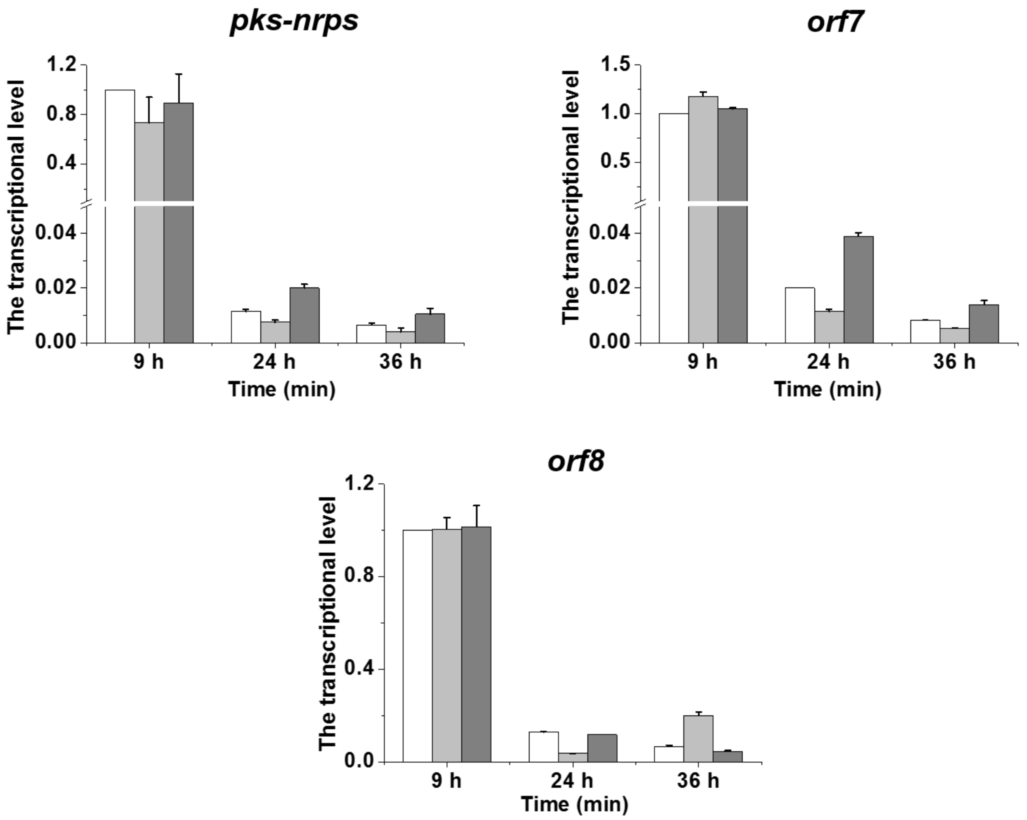

Figure S4. Transcriptional analysis of the HSAF biosynthetic genes, *pks-nrps*, *orf7* and *orf8*. The strains were cultured in 1/10 TSB medium. The white columns represent the gene transcriptional level in WT, the light gray columns represent the gene transcriptional level in ΔORF3232 strain, and the dark gray columns represent the gene transcriptional level in ORF3232CM strain. Data are presented as averages of three independent experiments, with each conducted in triplicate. *pks-nrps*, the structural gene of HSAF bisoynthesis; *orf7*, fatty acid hydroxylase gene; *orf8*, ferredoxin reductase gene.

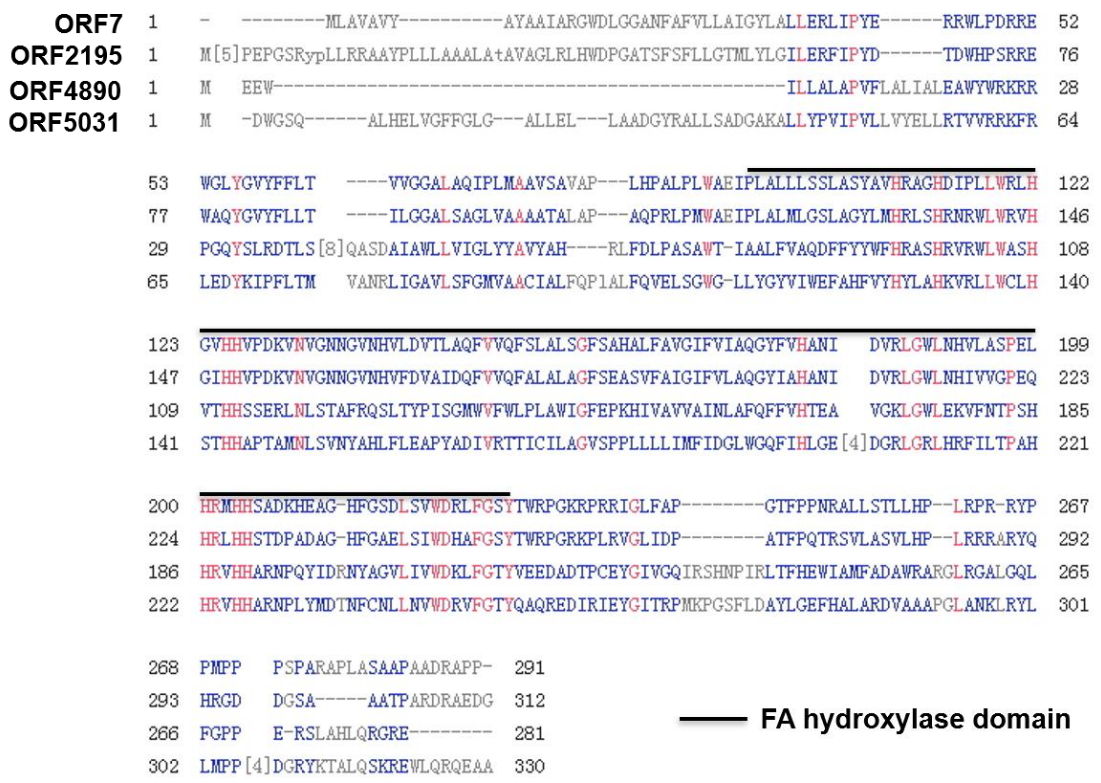

Figure S5. Alignment of the amino acid sequences of the known fatty acid hydroxylase gene (ORF7) with the putative fatty acid hydroxylases identified from the genome of OH11. The red color indicates identical columns and blue indicates high conserved ones.

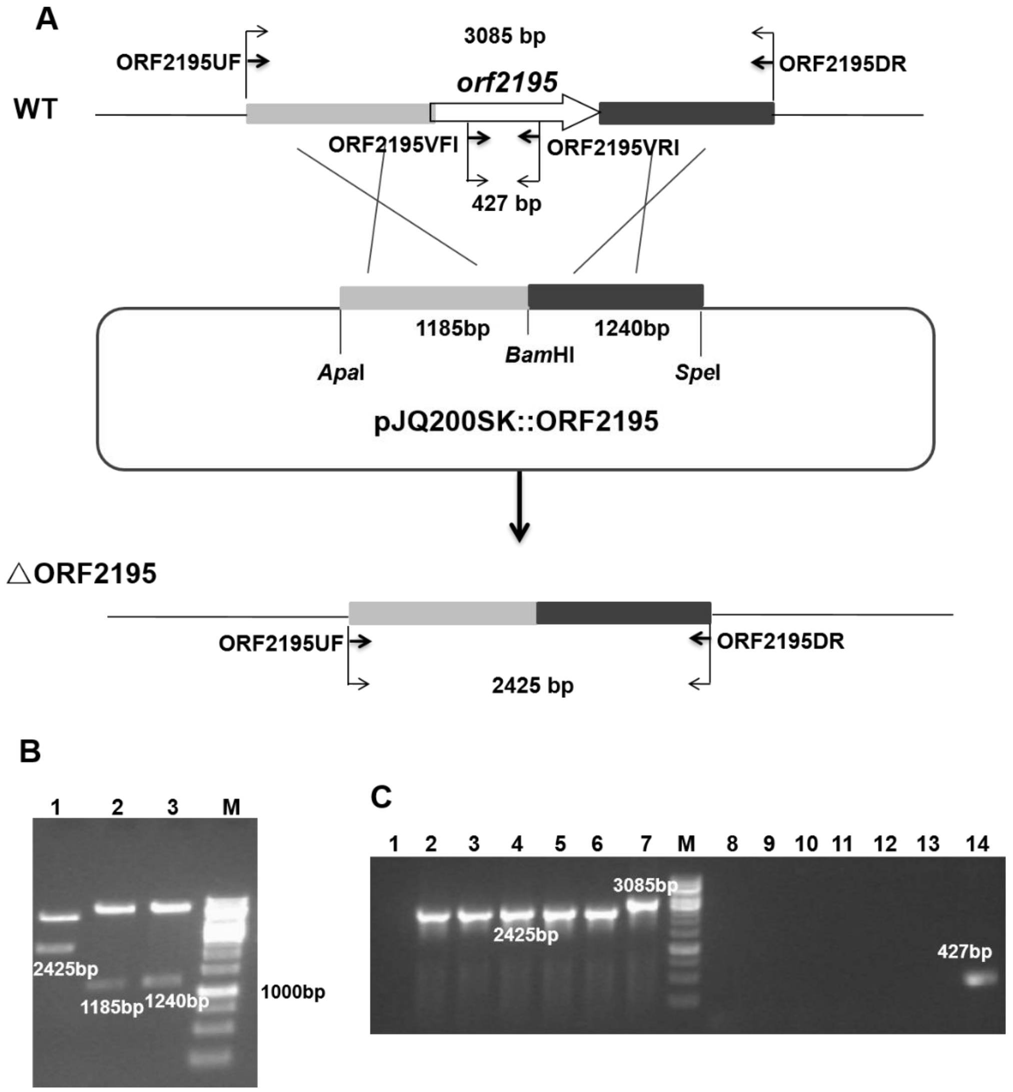

Figure S6. Deletion of *orf2195*. (A) Schematic representation of the *orf2195* deletion in WT strain. (B) Verification of the plasmid pJQ200SK::ORF2195. 1: pJQ200SK::ORF2195 treated with *Apa*I/*Spe*I, and the product of 2425 bp was expected; 2: pJQ200SK::ORF2195 treated with *Apa*I/*Bam*HI, and the product of 1185 bp was expected; 3: pJQ200SK::ORF2195 treated with *Bam*HI/*Spe*I, and the product of 1240 bp was expected; (C) PCR verification of ΔORF2195 strain. 1, 8: negative control, with H_2O used as the template; 2-6: the deletion mutant strains using primers ORF2195UF/DR, and the product of 2425 bp was expected; 7: positive control, with the gDNA of WT used as the template by primers ORF2195UF/DR, and the product of 3085 bp was expected; 9-13: the deletion mutant strains using primers ORF2195VFI/VRI; 10: positive control, with the gDNA of WT used as the template by primers ORF2195VFI/VRI, and the product of 427 bp was expected; M: DNA marker.

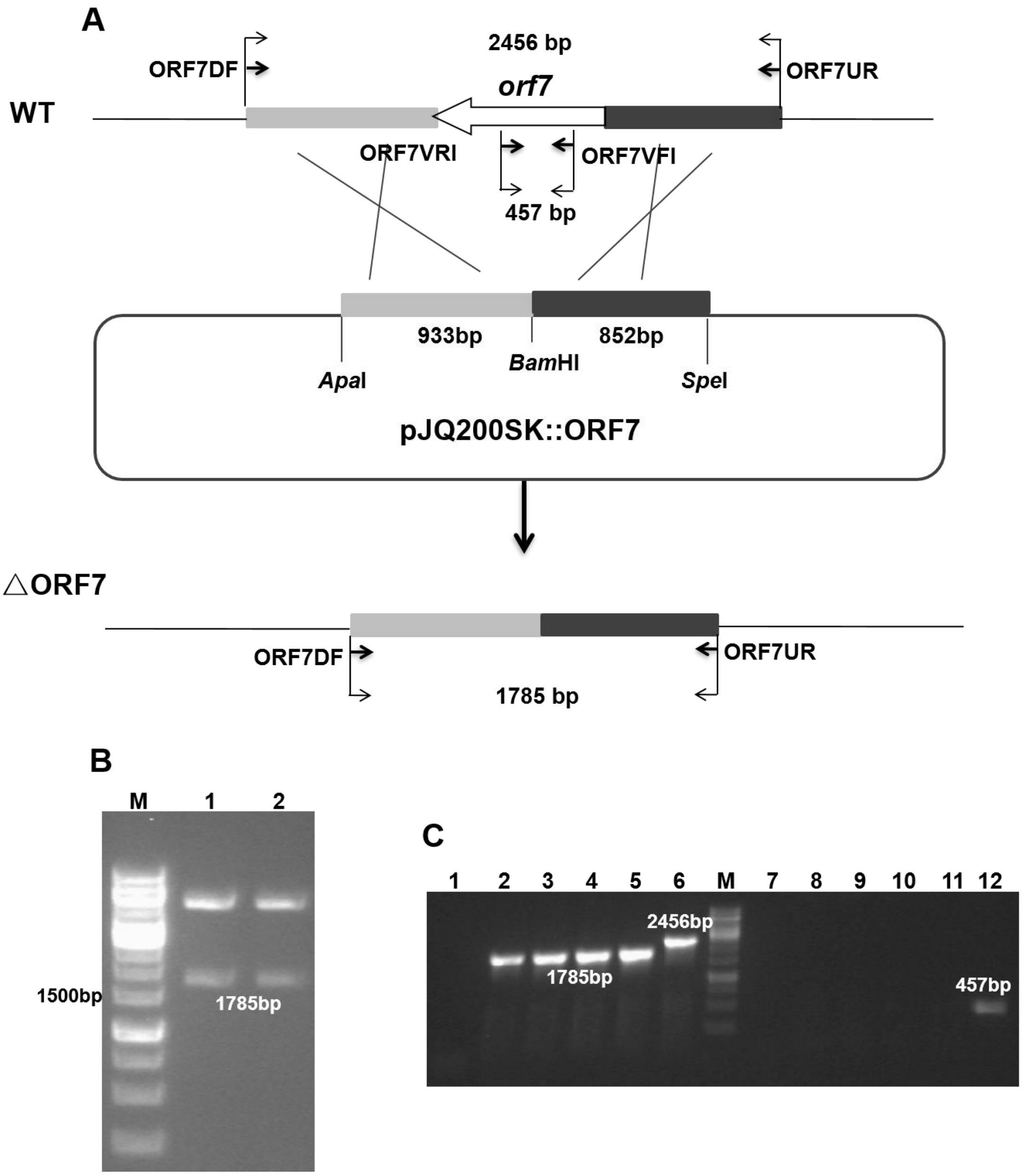

Figure S7. Deletion of *orf7* of the HSAF biosynthetic gene cluster. (A) Schematic representation of the *orf7* deletion in WT strain. (B) Verification of the plasmid pJQ200SK::ORF7. 1, 2: pJQ200SK::ORF7 treated with *Apa*I/*Spe*I, and the product of 1785 bp was expected. (C) PCR verification of ΔORF7 strain. 1, 7: negative control, with H_2O used as the template; 2-5: the deletion mutant strains using primers ORF7DF/UR, and the product of 1785 bp was expected; 6: positive control, with the gDNA of WT used as the template by primers ORF7DF/UR, and the product of 2456 bp was expected; 8-11: the deletion mutant strains using primers ORF7VFI/VRI; 12: positive control, with the gDNA of WT used as the template by primers ORF7VFI/VRI, and the product of 457 bp was expected; M: DNA marker.

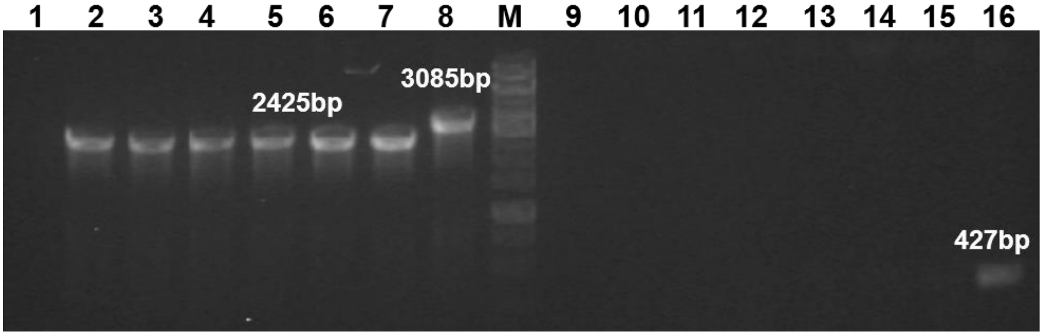

Figure S8. Deletion of both *orf7* and *orf2195*. 1, 9: negative control, with H_2O used as the template; 2-7: the double deletion mutant strains using primers ORF2195UF/DR, and the product of 2425 bp was expected; 8: positive control, with the gDNA of ΔORF7 strain used as the template by primers ORF2195UF/DR, and the product of 3085 bp was expected; 10-15: the double deletion mutant strains using primers ORF2195VFI/VRI; 16: positive control, with the gDNA of ΔORF7 strain used as the template by primers ORF2195VFI/VRI, and the product of 427 bp was expected; M: DNA marker.

Reference

1. Li, Y.; Huffman, J.; Li, Y.; Du, L.; Shen, Y., 3-Hydroxylation of the polycyclic tetramate macrolactam in the biosynthesis of antifungal HSAF from *Lysobacter enzymogenes* C3. *Med. Chem. Commun.* **2012**, 3, 982-987.
2. Qian, G.; Hu, B.; Jiang, Y.; Liu, F., Identification and characterization of *Lysobacter enzymogenes* as a biological control agent against some fungal pathogens. *Agric. Sci. China* **2009**, 8, (1), 68-75.
3. Quandt, J.; Hynes, M. F., Versatile suicide vectors which allow direct selection for gene replacement in gram-negative bacteria. *Gene* **1993**, 127, (1), 15-21.
